# Supplementary figures and images for: Revalidation of morphological characteristics and multiplex PCR for the identification of three congener invasive Liriomyza species (Diptera: Agromyzidae) in China
Source: PeerJ. 2020 Oct 30;8:e10138. doi: 10.7717/peerj.10138 (PMC7605219; doi:10.7717/peerj.10138)

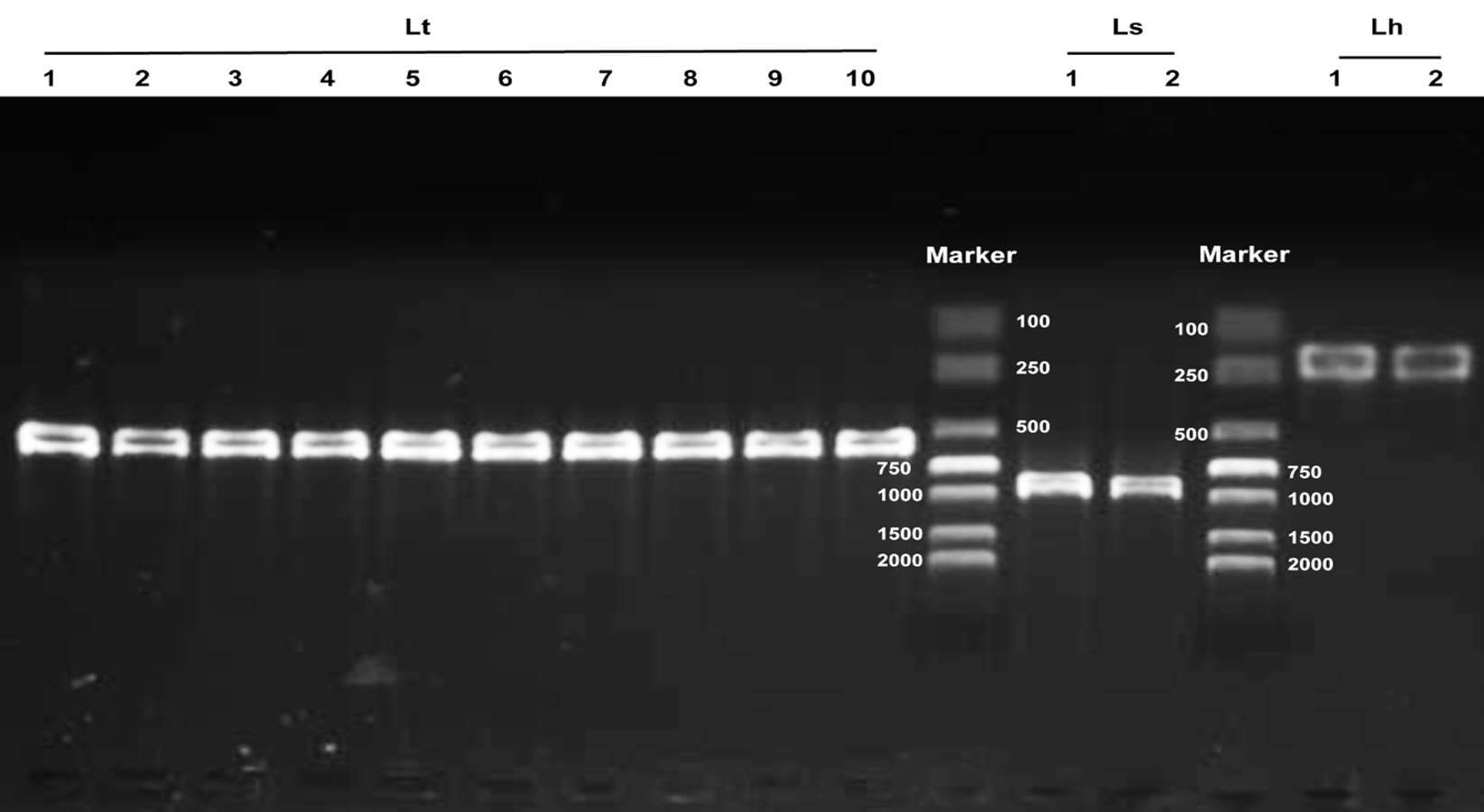

Supplement: Supplemental Information 2 — Lt Lanes 1-10 indicate L. trifolii populations from: (1) Hengshui, (2) Hangzhou, (3) Dongguan, (4) Zhangzhou, (5) Qionghai, (6), Nanning, (7) Changzhou, (8) Nanchang, (9), Huizhou, and (10) Huzhou. Ls lanes 1-2 indicate L. sativae populations from Shangqiu and Luoyang, respectively. Lh lanes 1-2 represent L. huidobrensis populations from Kunming and the laboratory, respectively. [file peerj-08-10138-s002.png]

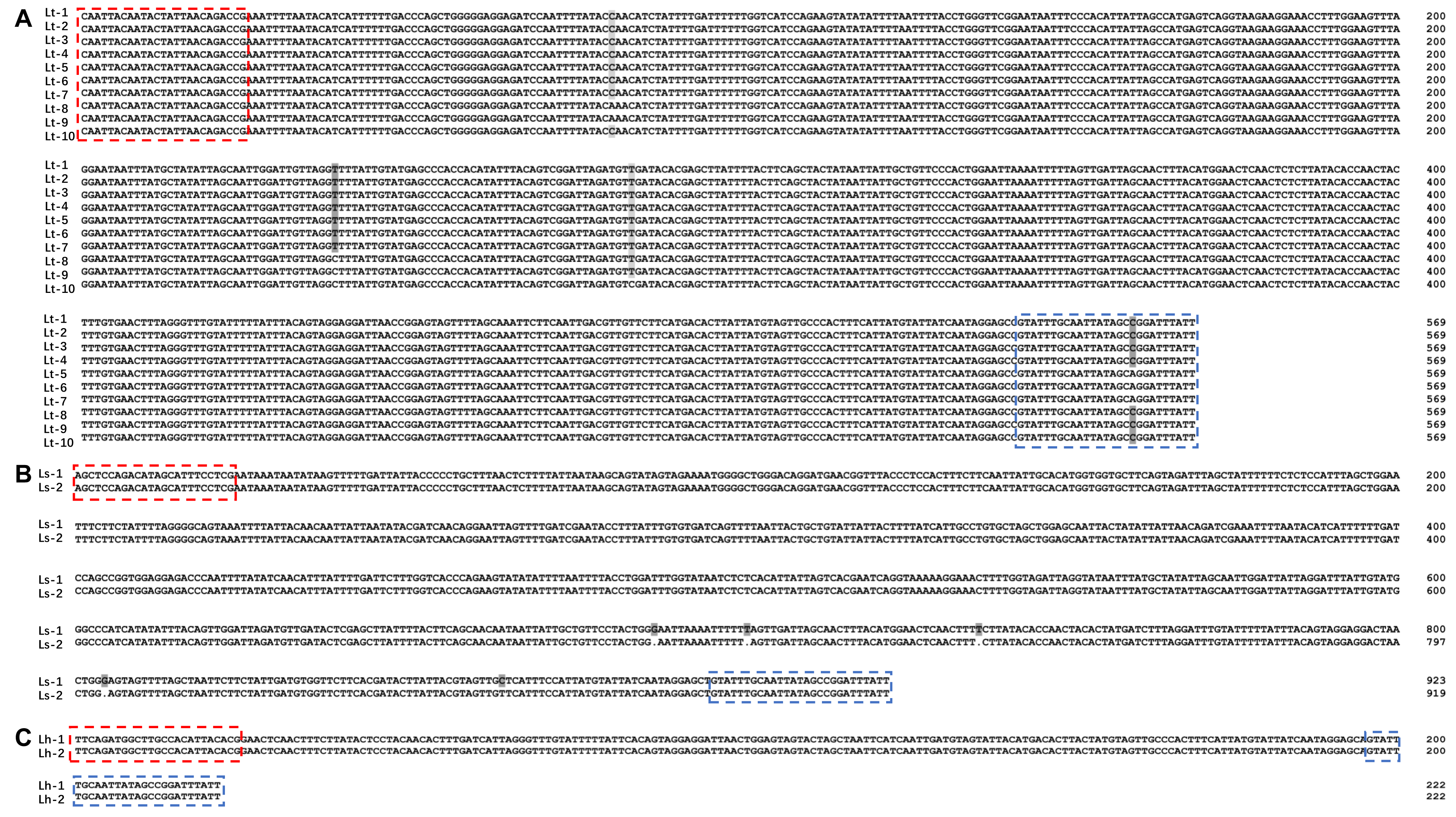

Supplement: Supplemental Information 3 — The shaded nucleotides represent divergent sites. Nucleotides bounded by red and blue dashed rectangles represent forward and reverse primers, respectively. Lt sequences represent the following L. trifolii populations: Lt 1, Hengshui; Lt 2, Hangzhou; Lt 3, Dongguan; Lt 4, Zhangzhou; Lt 5, Qionghai; Lt 6, Nanning; Lt 7, Changzhou; Lt 8, Nanchang; Lt 9, Huizhou; and Lt 10, Huzhou. Ls sequences represent L. sativae populations from Shangqiu (Ls 1) and Luoyang (Ls 2). Lh sequences represent L. huidobrensis populations from Kunming (Lh 1) and the laboratory (Lh 2). [file peerj-08-10138-s003.png]
