# Supplementary material for: Revalidation of morphological characteristics and multiplex PCR for the identification of three congener invasive Liriomyza species (Diptera: Agromyzidae) in China
Source: PeerJ. 2020 Oct 30;8:e10138. doi: 10.7717/peerj.10138 (PMC7605219; doi:10.7717/peerj.10138)
Supplement: Supplemental Information 4 [file peerj-08-10138-s004.docx]

**Table S1.** List of sample collection information

| Sample | Species | Collection location | Longitude  (°E) | Latitude (°N) | Host | Number | | Accession number |
| --- | --- | --- | --- | --- | --- | --- | --- | --- |
| SYJD | *L. trifolii* | Sanya | 109.51 | 18.25 | *Vigna unguiculata* | | 12 | MT932588-MT932599 |
| QHJC | *L. trifolii* | Qionghai | 110.47 | 19.26 | *Brassica juncea* | | 12 | MT932600-MT932611 |
| NNQC | *L. trifolii* | Nanning | 108.42 | 22.86 | *B. chinensis* | | 12 | MT932612-MT932623 |
| NNJD | *L. trifolii* | Nanning | 108.42 | 22.86 | *V. unguiculata* | | 10 | MT932624-MT932633 |
| HZQC | *L. trifolii* | Huizhou | 114.4 | 22.93 | *B. chinensis* | | 12 | MT932634-MT932645 |
| DGQC | *L. trifolii* | Dongguan | 113.74 | 23.01 | *B. chinensis* | | 12 | MT932646-MT932657 |
| DGJD | *L. trifolii* | Dongguan | 113.74 | 23.01 | *V. unguiculata* | | 12 | MT932658-MT932669 |
| WZJD | *L. trifolii* | Wuzhou | 111.23 | 23.43 | *V. unguiculata* | | 12 | MT932670-MT932681 |
| ZZJD | *L. trifolii* | Zhangzhou | 117.69 | 24.55 | *V. unguiculata* | | 12 | MT932682-MT932693 |
| KMQC | *L. huidobrensis* | Kunming | 103.28 | 25.52 | *Apium graveolens* | | 6 | MT926447- MT926452 |
| NCQC | *L. trifolii* | Nanchang | 115.91 | 28.67 | *A. graveolens* | | 12 | MT932694-MT932705 |
| HaZJD | *L. trifolii* | Hangzhou | 120.02 | 30.39 | *V. unguiculata* | | 12 | MT932706-MT932717 |
| WHJD | *L. trifolii* | Wuhan | 114.34 | 30.55 | *V. unguiculata* | | 12 | MT932718-MT932729 |
| HuZJD | *L. trifolii* | Huzhou | 119.99 | 31.04 | *V. unguiculata* | | 12 | MT932730-MT932741 |
| CZJD | *L. trifolii* | Changzhou | 119.9 | 31.63 | *V. unguiculata* | | 12 | MT932742-MT932753 |
| CSJD | *L. trifolii* | Changshu | 120.89 | 31.69 | *V. unguiculata* | | 12 | MT932754-MT932765 |
| SQJD1 | *L. trifolii* | Shangqiu | 115.7 | 34.51 | *V. unguiculata* | | 12 | MT932766-MT932777 |
| SQJD2 | *L. sativae* | Shangqiu | 115.7 | 34.51 | *V. unguiculata* | | 12 | MT926413- MT926424 |
| SQJD3 | *L. sativae* | Shangqiu | 115.7 | 34.51 | *Cucurbita moschata* | | 12 | MT926425- MT926436 |
| LYSG | *L. sativae* | Luoyang | 112.57 | 34.76 | *Luffa cylindrica* | | 10 | MT926437- MT926446 |
| HDJD | *L. trifolii* | Handan | 114.59 | 36.45 | *V. unguiculata* | | 9 | MT932778- MT932786 |
| HSJD | *L. trifolii* | Hengshui | 115.51 | 38 | *V. unguiculata* | | 12 | MT932787- MT932798 |
| HSFQ | *L. trifolii* | Hengshui | 115.51 | 38 | *Lycopersicon esculentum* | | 12 | MT932799- MT932810 |
